# A class of liquid anode for rechargeable batteries with ultralong cycle life

**DOI:** 10.1038/ncomms14629

**Published:** 2017-03-06

**Authors:** Juezhi Yu, Yong-Sheng Hu, Feng Pan, Zhizhen Zhang, Qing Wang, Hong Li, Xuejie Huang, Liquan Chen

**Affiliations:** 1 Key Laboratory for Renewable Energy, Beijing Key Laboratory for New Energy Materials and Devices, Beijing National Laboratory for Condensed Matter Physics, Institute of Physics, Chinese Academy of Sciences, School of Physical Sciences, University of Chinese Academy of Sciences, Beijing 100190, China; 2 Department of Materials Science and Engineering, National University of Singapore, Singapore 117576, Singapore

## Abstract

Low cost, highly efficient and safe devices for energy storage have long been desired in our society. Among these devices, electrochemical batteries with alkali metal anodes have attracted worldwide attention. However, the practical application of such systems is limited by dendrite formation and low cycling efficiency of alkali metals. Here we report a class of liquid anodes fabricated by dissolving sodium metal into a mixed solution of biphenyl and ethers. Such liquid anodes are highly safe and have a low redox potential of 0.09 V versus sodium, exhibiting a high conductivity of 1.2 × 10^−2^ S cm^−1^. When coupled with polysulfides dissolved in dimethyl sulfoxide as the cathode, a battery is demonstrated to sustain over 3,500 cycles without measureable capacity loss at room temperature. This work provides a base for exploring a family of liquid anodes for rechargeable batteries that potentially meet the requirements for grid-scale electrical energy storage.

## Abstract (web summary)

Ideal energy storage technologies should be efficient, safe and cost-effective. Here, the authors make progress by using dissolved sodium metal in a solution of biphenyl and ethers as a liquid anode for rechargeable sodium beta-alumina batteries.

One of the ‘Holy Grails' of rechargeable battery research is the successful application of alkali metals, such as lithium or sodium, as the anode to maximize the energy density utilizing their low negative potential and light weight^1^,^2^,^3^,^4^,^5^,^6^,^7^. However, the practical applications are hindered by the following formidable challenges. On the one hand, the alkali metals show poor cycling stability in organic liquid electrolytes because they are thermodynamically unstable with any kind of organic solvents. In addition, they tend to form dendrites during the deposition/stripping process, which increases the probability of internal short circuit, a well-known safety issue in real world applications^8^,^9^,^10^,^11^,^12^. On the other hand, even if the above-mentioned problems could be solved by replacing organic liquid electrolytes with solid electrolytes, other problems such as huge interfacial resistance between alkali metals and solid electrolytes could appear. As a result, molten or liquid alkali metals have been proposed, for example, in high-temperature sodium beta-alumina batteries, especially, the best-known sodium–sulfur (Na–S) and sodium–metal halide batteries. However, they need to be operated at a high temperature (300–350 °C) to decrease the interfacial resistance and to obtain better wettability between the alkali metals and solid electrolytes^13^,^14^,^15^,^16^,^17^. The high operating temperature is unfavourable because it gives rise to high corrosion rate, increases costs of battery manufacture and maintenance, as well as induces safety hazards. Actually, a few safety incidents have occurred in the last few years, which underscores the fact that safety issues of such systems have not been completely resolved and will limit their wide-scale application.

Here we report a liquid anode to enable such batteries to be operated at room temperature or moderate temperature. The liquid anode can be prepared simply by dissolving alkali metals into a solution of aromatic hydrocarbon and ethers. In particular, the example of sodium dissolved in biphenyl and dimethoxyethane (denoted as Na-BP-DME) is taken to demonstrate the strategy because of the natural abundance and wide geographic distribution of sodium resources^18^,^19^. The obtained liquid anodes possess a low potential of 0.09 V versus Na, a high conductivity of 1.2 × 10^−2^ S cm^−1^ at room temperature and are safer than Na metal anode. A rechargeable sodium beta-alumina battery is fabricated using the liquid anode and polysulfide cathode, presenting superior cycling performance at room temperature.

## Results

### Material synthesis and physical property

**Figure 1:**
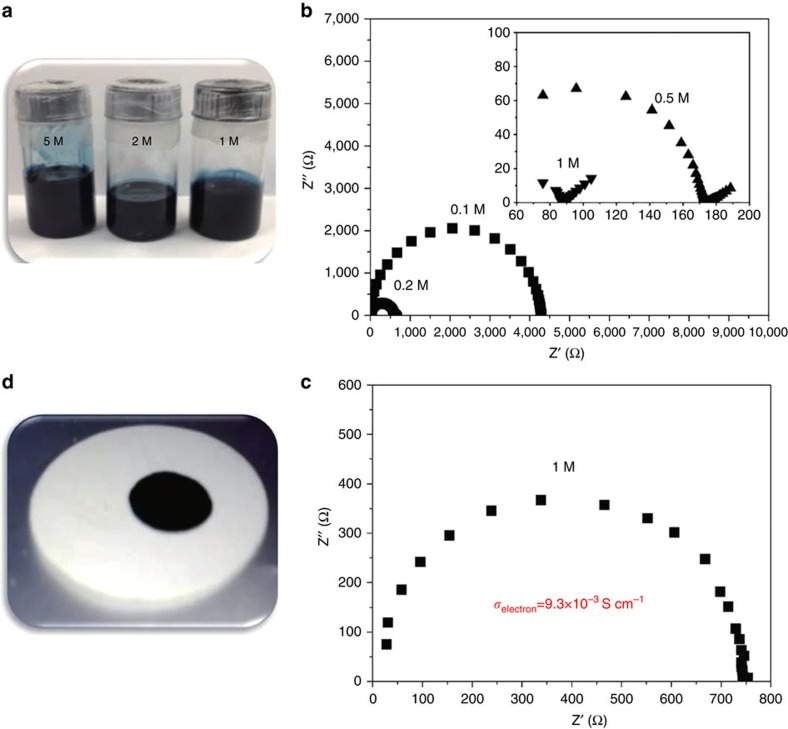
Physical properties of Na-BP-DME liquid solution. (**a**) Photograph of Na-BP-DME solution with different concentrations. (**b**) Electrochemical impedance spectra of Na-BP-DME solution with different concentrations (cell constant: *K*=1.05). (**c**) Electrochemical impedance spectrum of 1 M Na-BP-DME measured with Na^+^ ion blocking cell (cell constant: *K*=7.0). (**d**) Wetting behaviour of liquid Na-BP-DME drops on Na-β″-Al_2_O_3_ solid electrolyte.

The alkali metals, such as Li and Na, can react with some aromatic hydrocarbons in ether solvents to form a dark green alkali solution at room temperature. Such solutions have been known as radical anions and widely used as reducing agents in chemical synthesis since the 1930s^20^,^21^,^22^,^23^,^24^, however, they were not explored as electrodes for rechargeable batteries. In the following, the Na-BP-DME system is taken as an example to demonstrate the feasibility of using alkali solution as anodes for rechargeable batteries. First, let us describe the physical properties. The solubility of sodium and biphenyl or biphenyl in dimethoxyethane is revealed to be up to 5 M or 6 M, respectively, (Fig. 1a and Supplementary Fig. 1), which ensures a high volumetric energy density. Figure 1b shows the electrochemical impedance spectra of Na-BP-DME solutions with different Na concentrations (the molar ratio between Na and biphenyl was fixed at 1:1). It can be seen that the resistance decreases rapidly with the increase of Na concentration. At the concentration of 1 M, the total conductivity was calculated to be 1.2 × 10^−2^ S cm^−1^. To separate the electronic conductivity from the total conductivity, a special cell with a copper foil sandwiched between two Pt electrodes (Supplementary Fig. 2) was designed, which blocks Na^+^ ion transport during the measurement. The electrochemical impedance of the 1 M Na-BP-DME solutions was measured with an ion block cell and the spectrum is displayed in Fig. 1c, which help to calculate the electronic conductivity of 9.3 × 10^−3^ S cm^−1^. Hence, the ionic conductivity is 2.7 × 10^−3^ S cm^−1^. These values are sufficient for an electrode application.

The liquid electrode incorporated in the rechargeable battery has to be separated from the other electrode by a solid electrolyte. The wettability of this liquid electrode with the well-known Na-β″-Al_2_O_3_ solid electrolyte (BASE) was also checked. It can be seen that the Na-BP-DME liquid spreads over the entire surface of BASE at room temperature, suggesting a complete wetting of the surface with a contact angle of ∼0° (Fig. 1d and Supplementary Videos 1 and 2). This wettability is much superior to that of any other alkali metals or their alloys, even at a temperature as high as 200 °C (ref. 9). The superior stability of BASE in the Na-BP-DME solution was confirmed by the X-ray diffraction analysis (Supplementary Fig. 3).

### Electrochemical performance

**Figure 2:**
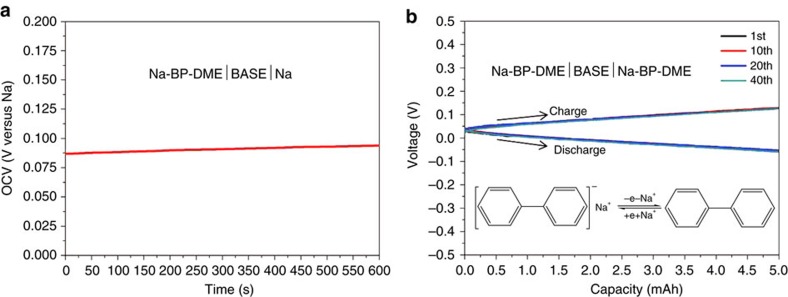
Electrochemical properties of Na-BP-DME liquid solution. (**a**) The open circuit voltage of Na-BP-DME|BASE|Na cell. (**b**) Cycle performance of Na-BP-DME|BASE|Na-BP-DME symmetric cell.

To determine the redox potential of the Na-BP-DME liquid solution, a Na-BP-DME|BASE|Na cell was fabricated. The open circuit voltage curve of the cell is presented in Fig. 2a and shows a redox potential of 0.09 V versus Na, which is very close to the redox potential of Na metal, making this liquid suitable as an anode in rechargeable batteries. This low potential is also beneficial to maximize the energy density of a battery. The electrochemically reversible Na removal/uptake from this liquid solution was also demonstrated in a symmetric Na-BP-DME|BASE|Na-BP-DME cell and the corresponding electrochemical reactions at the cathode and anode are shown as equation (1).

The concentration of both electrodes was 1 M. The charge/discharge capacity was fixed at 5 mAh for a 0.43 ml liquid cathode and anode, corresponding to 0.5 mol Na removal/uptake per 1 mol Na-BP-DME (named as 0.5Na-BP-DME and 1.5Na-BP-DME, respectively). Figure 2b indicates that this process is highly reversible. From this result, Na-BP-DME elecrtode can be charged/discharged between 0.5Na-BP-DME and 1.5Na-BP-DME (note that the conductivities of 0.5Na-BP-DME and 1.5Na-BP-DME liquid solutions remain as high as 5.8 × 10^−3^ S cm^−1^ and 1.4 × 10^−2^ S cm^−1^, respectively, (Supplementary Fig. 4 and Supplementary Table 1)).

**Figure 3:**
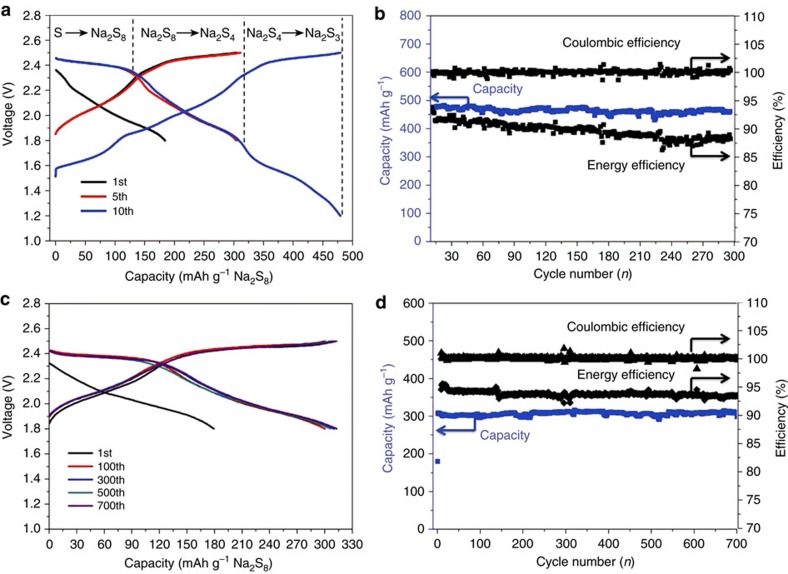
Electrochemical performance of Na_2_S_8_|BASE|Na-BP-DME (1 M) cells. (**a**) Galvanostatic discharge and charge profiles of Na_2_S_8_|BASE|Na-BP-DME cells cycled between 1.2 and 2.5 V. (**b**) Cycle stability of Na_2_S_8_|BASE|Na-BP-DME cells in the voltage range of 1.2–2.5 V under a constant current density of 300 mA g^−1^. (**c**) Galvanostatic discharge and charge profiles of Na_2_S_8_|BASE|Na-BP-DME cells cycled between 1.8 and 2.5 V. (**d**) Cycle performance of Na_2_S_8_|BASE|Na-BP-DME cells in the voltage range of 1.8–2.5 V under a constant current densities of 300 mA g^−1^. These cells were assembled with Na-β″-Al_2_O_3_ tube (L50 × OD13 mm, 1 mm thickness).

Based on these unique characteristics, we are able to construct a rechargeable battery using this liquid anode. Here we selected the liquid sulfur-based cathode to demonstrate the practical application of room temperature sodium beta-alumina batteries. The liquid cathode was prepared by dissolving S and Na_2_S with a molar ratio of 7:1 into dimethyl sulfoxide (DMSO) solvent. The concentration of Na_2_S_8_ in DMSO can be up to 1 M (Supplementary Fig. 5a), ensuring a high volumetric energy density^7^. A prototype Na_2_S_8_|BASE|Na-BP-DME cell was assembled with a BASE tube separating the liquid anode (inside the tube) and liquid cathode, and the carbon felt and nickel foam were used as the current collectors, respectively, (Supplementary Fig. 6a,b). The cell was first discharged to 1.8 V and then charged to 2.5 V at a current density of 300 mA g^−1^ (that is, 1C rate). After several cycles between 1.8 and 2.5 V, the cutoff voltage was set between 1.2 and 2.5 V. Typical discharge and charge profiles are presented in Fig. 3a. The discharge profile displays three different regions, which corresponds to S-to-Na_2_S_8_ (I), Na_2_S_8_-to-Na_2_S_4_ (II) and Na_2_S_4_-to-Na_2_S_3_ (III), respectively.

The high reversibility of those three processes is manifested by over 300 cycles with capacity retention of 95% (Fig. 3b). Although the cycling stability is good, the round-trip efficiency is only 90% in the initial cycles and stabilizes at 87%, which is mainly due to the sluggish kinetics^9^ and the limited solubility of Na_2_S_3_ in DMSO (Supplementary Fig. 5b). To further increase the round-trip efficiency, the cutoff voltage is set between 1.8 and 2.5 V (Fig. 3c). The electrochemical reactions at the anode and cathode are:

Anode:


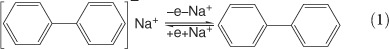


Cathode:


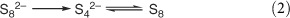


It can be seen that the reversible capacity in this voltage range is 310 mAh g^−1^, which corresponds to the formation of Na_2_S_4_ that can be fully dissolved in DMSO (Supplementary Fig. 5c). It is noteworthy that the cell exhibits outstanding cycling performance with no measureable capacity loss after 700 cycles (Fig. 3d), suggesting superior stability of both sulfur cathodes and Na-BP-DME liquid anodes. The Coulombic efficiency is close to 100%, resulting from the use of a solid electrolyte, which blocks the diffusion of polysulfide to the anode side. Most importantly, the round-trip efficiency remains at 94% after 700 cycles. The volumetric and gravimetric energy densities of the system with this liquid anode and polysulfide cathode were calculated to be 94 Wh l^−1^ and 80 Wh kg^−1^, respectively, (Supplementary Tables 2 and 3) (note that the 5 M Na-BP-DME system displays the very similar discharge/charge behaviour to that of 1 M Na-BP-DME system as shown in Supplementary Fig. 7 under the same conditions.). The cost of raw materials in this system is only 11.8$ kWh^−1^ (Supplementary Table 4, without considering the cost of BASE.). These values are superior to conventional redox flow batteries^25^ and higher than recently reported new battery systems^26^,^27^,^28^,^29^,^30^ (Supplementary Table 5). Note that, in principle, we can even add Na metal into this saturated Na-BP-DME solution to achieve much higher energy density (201 Wh l^−1^ and 149 Wh kg^−1^ (Supplementary Table 3)). In this case, the liquid can function as an interfacial wetting agent between solid electrolyte and Na metal (Supplementary Fig. 6c). The electrochemical performance of Na metal in Na-saturated BP-DME liquid hybrid electrode is shown in Supplementary Fig. 8. It can be seen that this hybrid electrode displays a high reversibility and stability.

**Figure 4:**
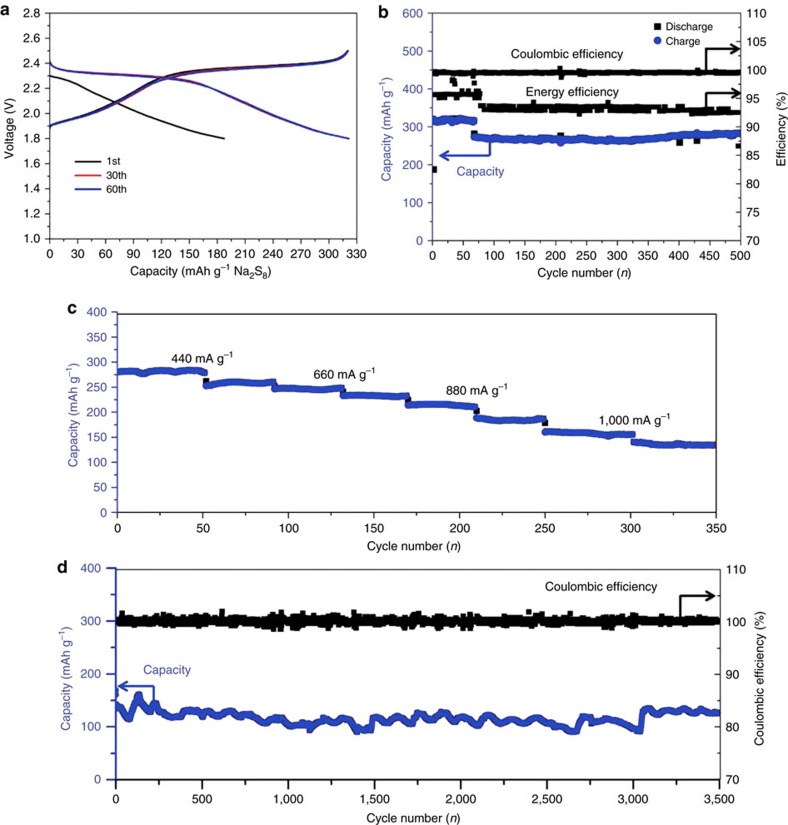
Electrochemical performance of Na_2_S_8_|BASE|Na-BP-TEGDME (1 M) cells. (**a**) Galvanostatic discharge and charge profiles of Na_2_S_8_|BASE|Na-BP-TEGDME cells cycled between 1.8 and 2.5 V. (**b**) Cycle stability of Na_2_S_8_|BASE|Na-BP-TEGDME cells in the voltage range of 1.8–2.5 V under a constant current density of 330 and 440 mA g^−1^. (**c**) Rate performance of Na_2_S_8_|BASE|Na-BP-TEGDME cells cycled between 1.8 and 2.5 V at different current density. (**d**) Long cycle stability of Na_2_S_8_|BASE|Na-BP-TEGDME cells in the voltage range of 1.8–2.5 V under constant current densities of 1,100 mA g^−1^. These cells were assembled with Na-β″-Al_2_O_3_ tube (L50 × OD13 mm, 1 mm thickness).

In this alkali metal solution, other ether solvents can also be used. The conductivities of all the tested systems are in the order of 10^−3^ S cm^−1^ (Supplementary Fig. 9). In particular, the electrochemical performance of Na_2_S_8_|BASE|Na-BP-TEGDME cell was demonstrated to be similar to that of Na-BP-DME system (Fig. 4a,b). The rate performance of Na_2_S_8_|BASE|Na-BP-TEGDME cell is shown in Fig. 4c and it can be seen that the capacity retention of 63% at 880 mA g^−1^ can be achieved without optimization. Furthermore, the cell was cycled at 1,100 mA g^−1^ for 3,500 cycles without any capacity decay (Fig. 4d).

Another noteworthy property of this liquid anode is the high degree of safety. It is well known that Na metal reacts drastically with water, posing a serious safety hazard. Here we checked the reaction of Na-BP-DME liquid anode by adding water drop by drop into the Na-BP-DME solution. Interestingly, the reaction is much milder than that of Na metal and no fire was observed during the whole process (Supplementary Fig. 10a and Supplementary Videos 3 and 4). This is because the reaction follows as refs 15, 16, 17:


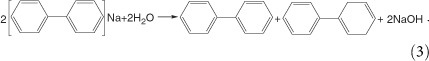


Unlike Na metal reacting with water (Supplementary Video 5), no hydrogen (H_2_) is generated during the reaction. Furthermore, no drastic reaction occurred when adding Na_2_S_8_ solution into the Na-BP-DME solution drop by drop (Supplementary Fig. 10c and Supplementary Video 6). The change of temperature for both reactions was also recorded and shown in Supplementary Fig. 10b,d. It can be seen that the increase of temperature cannot exceed 20 °C when Na-BP-DME reacts with water, while the temperature change is less than 10 °C for the reaction of Na-BP-DME with Na_2_S_8_ solution. These peculiar properties make the room temperature Na_2_S_8_|BASE|Na-BP-DME battery intrinsically safer than the state-of-the-art high-temperature Na–S batteries, which is a very important advantage by using this system for large-scale applications.

## Discussion

In conclusion, we have demonstrated the use of a class of Na-BP-Ether liquid anode in room temperature rechargeable sodium beta-alumina batteries. The liquid anodes hold some distinct advantages over the current alkali metal-based technologies. First, this liquid anode has high electronic/ionic conductivity with superior reversibility and stability of Na removal/uptake at a low redox potential. It can wet the solid electrolyte well, and is dendrite-free and SEI-free with low cost and high safety. Second, our strategy is tunable as different alkali metals, aromatic hydrocarbons or ether solvents can be chosen to tune the physical and electrochemical properties^13^,^14^. Third, sulfur cathodes, and other high soluble^30^ or slurry cathodes can also be used^31^. Finally, the cell can be designed as either a cylindrical/planar cell or a redox flow cell with a separation of energy and power as shown in Supplementary Fig. 11 (note that the preliminary electrochemical performance of the redox flow battery at this stage was shown in Supplementary Figs 12 and 13. We believe that the performance can be further improved by optimizing the system and engineering the cell structure, for instance, using new catholyte system with higher energy density, a thinner Na-β″-Al_2_O_3_ electrolyte or new electrolyte with higher ionic conductivity, and a highly porous current collector). This class of liquid anode could open up an exciting route for long-life, cost-effective and safe rechargeable batteries that potentially meet the requirements for grid-scale electrical energy storage.

## Methods

### Preparation of Na-BP-DME liquid anodes

A dark green liquid Na-BP-DME was prepared by dissolving sodium metal (Aldrich, 99.5%) into biphenyl (Aldrich, 99%) and DME (BASF) solution in glove box filled with Argon. In a typical procedure, 1.54 g biphenyl was dissolved into 10 ml DME solvent to make a 1 M BP-DME solution and then 0.23 g sodium metal was dissolved into this solution to form a 1 M Na-BP-DME solution. It should be noted that the molar ratio of Na:BP was fixed at 1:1. Different concentrations of Na-BP-DME or Na-BP-TEGDME liquid solutions were prepared by the same procedure.

### Electronic and Na^+^ ionic conductivity measurement

Total conductivity was measured by the electrochemical impedance spectrum, which was carried out on a Zahner IM6. The test frequency ranges from 100 mHz to 6 MHz. The conductance cell is Rosull DJS-1(cell constant: *K*=1.05) as displayed in Supplementary Fig. 2a. A Na^+^ ion blocking cell which was illustrated in Supplementary Fig. 2b was used to measure the electronic conductivity of Na-BP-DME liquid solution (cell constant: *K*=7.0). The Na^+^ ion can be blocked by the copper foil (thickness: 8 μm) in the ion blocking cell, but electron can pass through the whole cell during measurement.

### Preparation of Na_2_S_8_-DMSO liquid cathodes

The Na_2_S_8_ liquid cathode was prepared in glove box filled with Argon. Typically, 0.78 g Na_2_S (Aldrich, 98%), 2.24 g S (Aldrich, 99%) and 0.85 g CF_3_SO_3_Na (Aldrich, 99.5%) were dissolved into 10 ml DMSO solvent to form a 1 M Na_2_S_8_-DMSO solution (note that NaSO_3_CF_3_ was used as the supporting electrolyte).

### Cell construction and electrochemical tests

Single cells of Na_2_S_8_|BASE|Na-BP-DME were assembled according to a high temperature Na–S battery. In detail, 1 ml 1 M Na-BP-DME solution was injected into Na-β″-Al_2_O_3_ tube (L50 × OD13 mm, 1 mm in thickness, Ionotec Company, ionic conductivity at room temperature is 1.67 × 10^−3^ S cm^−1^) and nickel foam was plugged into this solution as a current collector. Then, Na_2_S_8_-DMSO liquid cathode was poured into stainless steel cylinder housing (L60 × OD18 mm) and carbon felt was used as a current collector. Finally, the Na-β″-Al_2_O_3_ tube filled with Na-BP-DME solution was inserted into the stainless steel cylinder filled with Na_2_S_8_-DMSO liquid cathode. Electrochemical performance was tested with BT-2000 Arbin Battery Testing system at room temperature. The cells were discharged and charged in the voltage ranges of 1.2–2.5 V and 1.8–2.5 V at a constant current mode.

Symmetric cell of Na-BP-DME|BASE|Na-BP-DME was assembled with 1 M Na-BP-DME as cathode and anode. In detail, 0.43 ml 1 M Na-BP-DME solution was injected into Na-β″-Al_2_O_3_ tube and stainless steel cylinder, nickel foam was plugged into this solution as a current collector. Electrochemical performance was tested with BT-2000 Arbin Battery Testing system at room temperature with a limited capacity of 5 mAh. The same symmetric cell with excess Na metal soaked in Na-BP-DME was assembled as the same procedure, the only difference is that excess Na metal piece was added into the Na-BP-DME cathode and anode (note that the concentration of BP in DME was 1 M). Electrochemical performance was tested with BT-2000 Arbin Battery Testing system at room temperature with a limited capacity of 6 mAh.

### Reaction temperature tests

The changes of temperature for reactions of Na-BP-DME with water and Na_2_S_8_ cathode were measured with highly precise temperature sensor JULABO-PHYSICS (Julabo Company). In detail, the temperature sensor was inserted into the inside drop by drop added 1 ml Na-BP-DME and then 1 ml water. Similarly, the temperature sensor was also inserted into the inside dropwise added 40 ml Na-BP-DME and then 10 ml Na_2_S_8_-DMSO.

### Data availability

The authors declare that the data supporting the findings of this study are available within the article and its Supplementary Information Files. All other relevant data supporting the findings of this study are available from the corresponding author on request.

## Additional information

**How to cite this article:** Yu, J. *et al*. A class of liquid anode for rechargeable batteries with ultralong cycle life. *Nat. Commun.* **8,** 14629 doi: 10.1038/ncomms14629 (2017).

**Publisher's note:** Springer Nature remains neutral with regard to jurisdictional claims in published maps and institutional affiliations.

## Supplementary Material

Supplementary Information Supplementary Figures 1-13, Supplementary Tables 1-5, Supplementary Methods and Supplementary References

Supplementary Movie 1 Wettability of 1 M Na-BP-DME

Supplementary Movie 2 Wettability of 5 M Na-BP-DME

Supplementary Movie 3 1 M Na-BP-DME react with water

Supplementary Movie 4 4 M Na-BP-DME react with water

Supplementary Movie 5 Na metal reacts with water

Supplementary Movie 6 Na-BP-DME reacts with Na2S8-DMSO

Supplementary Movie 7 Flowability of 5 M Na-BP-DME

Supplementary Movie 8 Flow battery
