# Supplementary material for: A class of liquid anode for rechargeable batteries with ultralong cycle life
Source: Nat Commun. 2017 Mar 6;8:14629. doi: 10.1038/ncomms14629 (PMC5343454; doi:10.1038/ncomms14629)
Supplement: Supplementary Information — Supplementary Figures 1-13, Supplementary Tables 1-5, Supplementary Methods and Supplementary References [file ncomms14629-s1.pdf]

Supplementary Figures

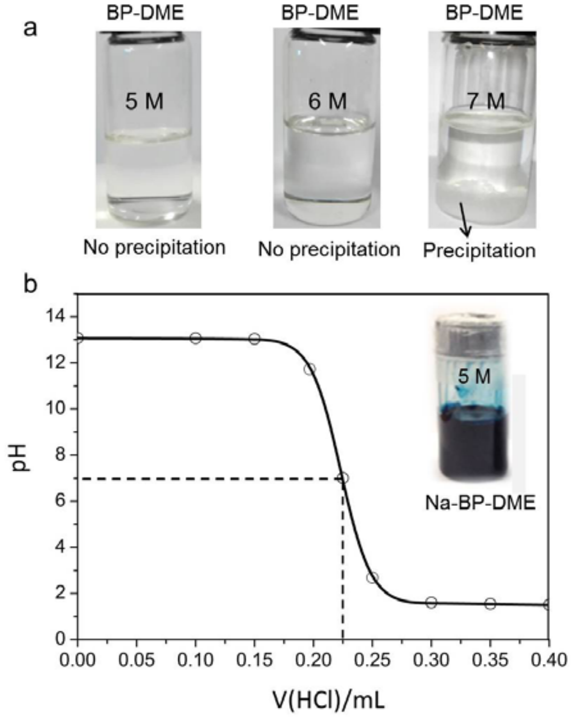

Supplementary Figure 1 | Solubility of BP in DME and Na in BP-DME solvent. a, Photograph of BP-DME solutions. **b,** Chemical titration profile and photograph of 5 M Na-BP-DME solution. Chemical titration was carried out to check solubility of Na in BP-DME solvent. In detail, 1 mL Na saturated 5 M Na-BP-DME solution was dissolved into 3 mL H_2O, and the final products (NaOH solution) was titrated by 0.225 mL HCl

to PH=7.0. From this result, we can calculate the solubility of Na is 5 M.

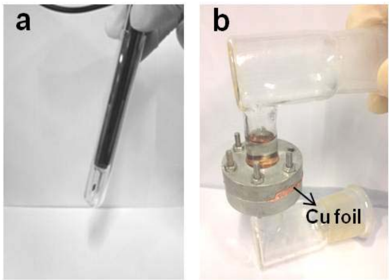

Supplementary Figure 2 | Photograph of conductivity measurement devices. a, Illustration of Rosull DJS-1 cell, the cell constant is K=1.05. **b**, Illustration of the Na^+ ion blocking cell which was designed by sandwiched a copper foil between two Pt electrodes to block Na^+ ion transportation during measurement, the cell constant is K=7.0.

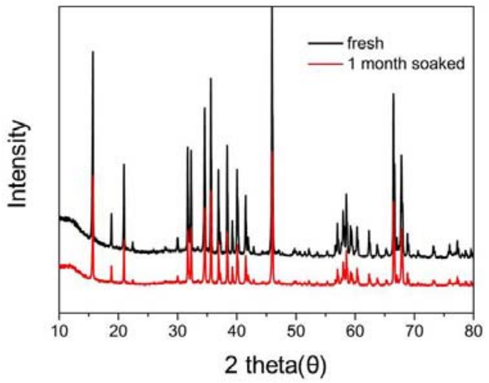

Supplementary Figure 3 | Stability of Na-β"-Al_2O_3 in Na-BP-DME solution. XRD patterns of fresh Na-β"-Al_2O_3 compared with Na-β"-Al_2O_3 soaked in Na-BP-DME for one month.

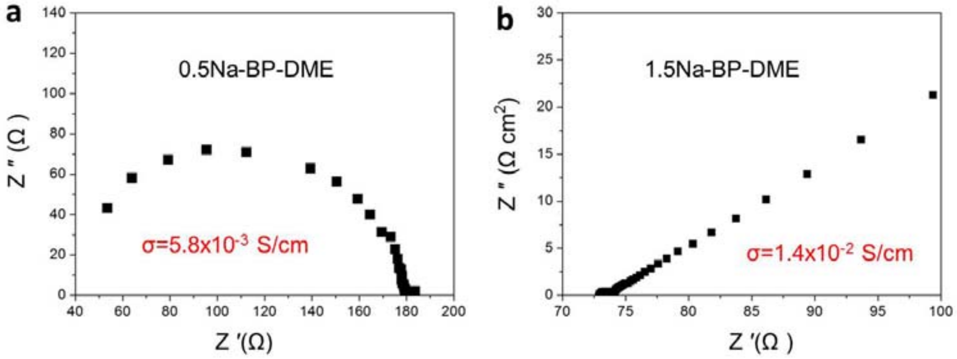

Supplementary Figure 4 | Electrochemical impedance spectra. **a,** Electrochemical impedance spectrum of 1 M 0.5Na-BP-DME (cell constant K=1.05) (Note that the

0.5Na-BP-DME refers to 0.5 mol Na removal from 1 mol Na-BP-DME.). **b,** Electrochemical impedance spectrum of 1 M 1.5Na-BP-DME (cell constant K=1.05) (Note that the 1.5Na-BP-DME refers to 0.5 mol Na uptake into 1 mol Na-BP-DME.).

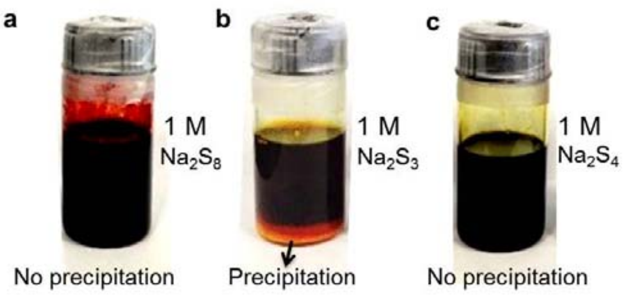

Supplementary Figure 5 | Solubility of polysulfides in DMSO. a, Photograph of Na_2S_8 dissolve into DMSO solutions. **b,** Photograph of Na_2S_3 dissolve into DMSO solutions. **c,** Photograph of Na_2S_4 dissolve into DMSO solutions. Na_2S_8, Na_2S_4 and Na_2S_3 solutions were prepared by dissolving Na_2S and S into DMSO at molar ratio of 1:7, 1:3, 1:2 respectively, and the concentrations of Na_2S_8, Na_2S_4 and Na_2S_3 were 1 M respectively. For 1 M Na_2S_3 system, one can clearly see that there is precipitation on the bottom, indicating that the solubility of Na_2S_3 in DMSO is less than 1 M. In contrast, we do not observe any precipitation for 1 M Na_2S_4 and Na_2S_8 system, which is in good agreement with Ref. [7].

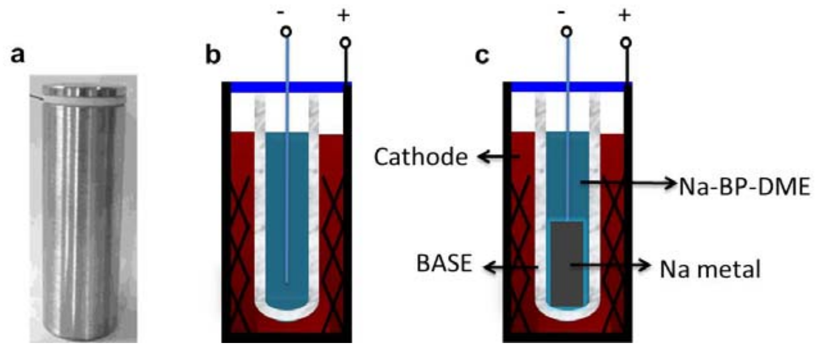

Supplementary Figure 6 | Photograph and schematic of the cylinder cell. **a,** The stainless steel cylinder cell. **b,** Schematic of the Na_2S_8 | BASE | Na-BP-DME cell in which nickel foam was used as the Na-BP-DME anode current collector and carbon felt as the Na_2S_8 cathode current collector. **c,** Schematic of the Na_2S_8 | BASE | Na-BP-DME cell with Na metal insertion into Na saturated BP-DME hybrid anode.

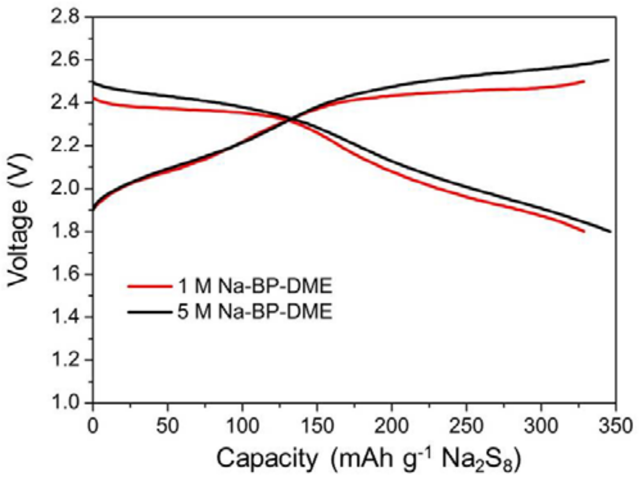

Supplementary Figure 7 | Electrochemical performance of the cell with high concentration Na-BP-DME liquid anode. Typical charge-discharge profiles of the cell with 1 M Na-BP-DME and 5 M Na-BP-DME anode.

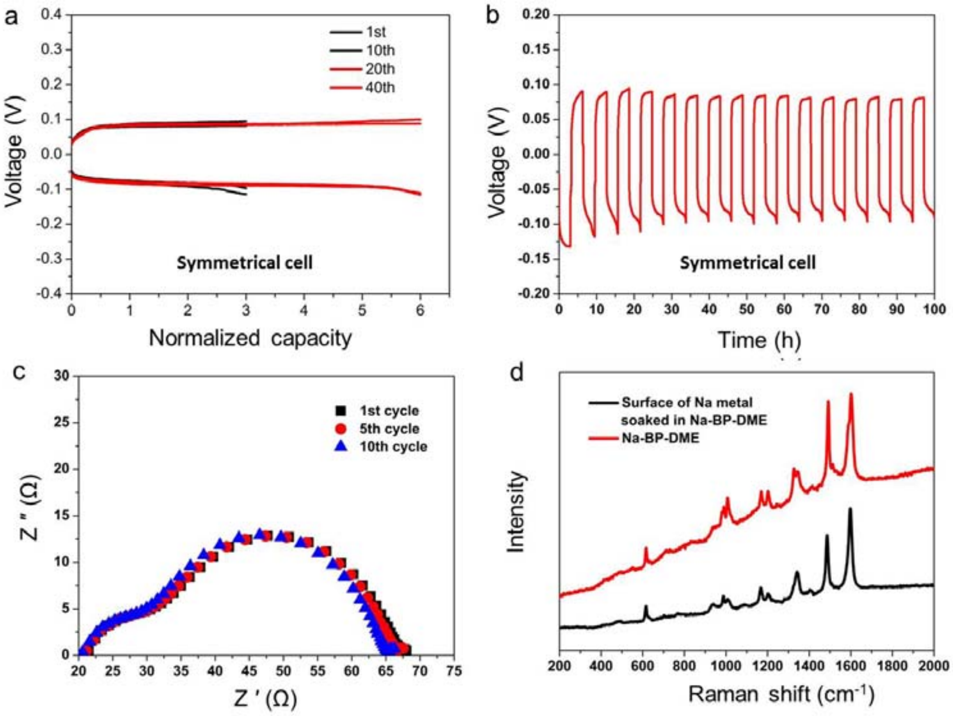

Supplementary Figure 8 | Electrochemical performance of Na saturated Na-BP-DME solution with an excessive Na metal soaked inside. a, Charge-discharge profiles of symmetric cell constructed with Na saturated Na-BP-DME solution as cathode and anode at a constant current of 0.5 mA. **b,** Voltage vs. time profiles of this symmetric cell. **c,** The electrochemical impedance spectra of this symmetric cell along with different cycles. **d,** Raman spectra of Na saturated Na-BP-DME solution and Na metal taken from the solution. These preliminary results shown here indicate

that this Na saturated Na-BP-DME solution exhibits a high Na^+ uptake and removal reversibility and stability. The Raman results show that the components from the surface of Na metal are the same as the Na saturated Na-BP-DME solution, suggesting that there is no SEI formation on Na metal surface when Na is soaked in this solution.

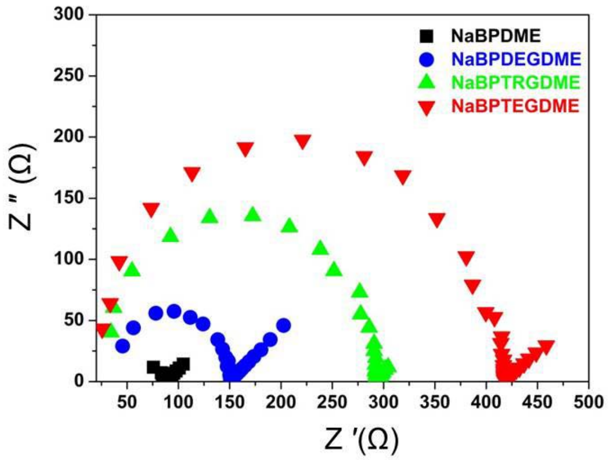

Supplementary Figure 9 | Conductivity of Na-BP-ether systems. Electrochemical impedance spectra of Na-BP dissolved in different ether solvents: DME, DEGDME,

TRGDME, and TEGDME. There conductivities at room temperature are calculated to be $1.2x10^{-2}$ S cm^{-1}, $7x10^{-3}$ S cm^{-1}, $3.5x10^{-3}$ S cm^{-1}, $2.5x10^{-3}$ S cm^{-1}, respectively (cell's constant K=1.05).

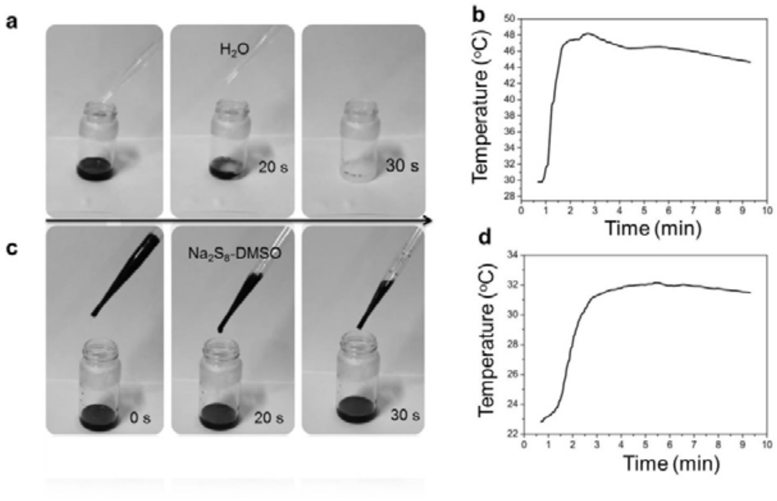

Supplementary Figure 10|Safety tests of Na-BP-DME liquid anode. **a**, Reaction of Na-BP-DME liquid anode with distilled water. **b,** Temperature change profile of 1 mL Na-BP-DME react with 1 mL water. **c**, Reaction of Na-BP-DME liquid anode with Na_2S_8-DMSO liquid cathode. **d,** Temperature change profile of 40 mL Na-BP-DME (1M) react with 10 mL Na_2S_8-DMSO (1 M) cathode (corresponding to ca. 500 mAh cell).

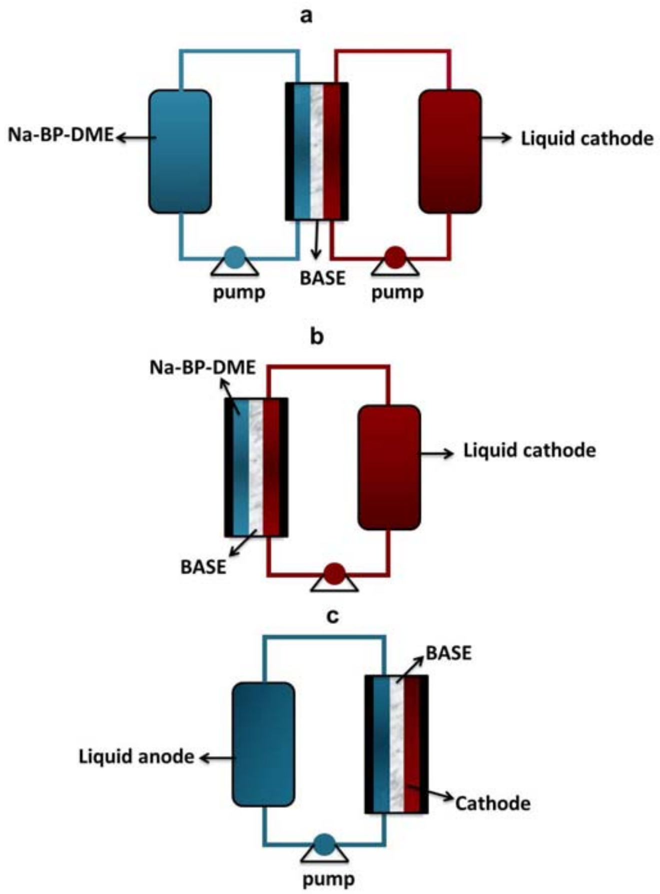

Supplementary Figure 11 | Schematic of the redox flow cells with Na-BP-DME liquid anode. **a**, Schematic of common redox flow cell . **b,** Schematic of single cathode flow cell. **c,** Schematic of single anode flow cell.

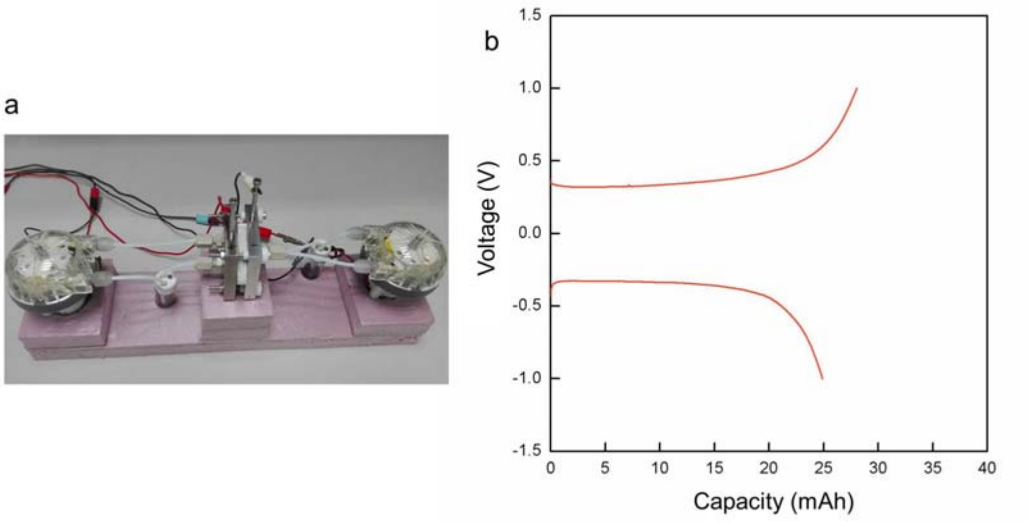

Supplementary Figure 12 | Electrochemical performance of symmetrical redox flow battery. **a,** Digital photograph of the designed redox flow battery operated in the glove box, the thicknesses of the BASE and the electrode are 2 mm and 10 mm respectively. **b,** Typical charge-discharge profile of (3 M) Na-BP-TEGDME | BASE | Na-BP-TEGDME (0.5 M) symmetrical cell. Symmetrical cell was constructed with Na (3 M) dissolved into 3 M BP-TEGDME as catholyte, and Na (0.5 M) dissolved into 3 M BP-TEGDME as anolyte. This cell was named as 3Na-BP-TEGDME | BASE | 0.5Na-BP-TEGDME. This cell was assembled to investigate the reversibility of 3 M Na-BP-TEGDME. The volume of each electrolyte was 0.58 mL, theoretical capacity is 33 mAh based on volumetric capacity of 3 M Na-BP-TEGDME. Then, the utilization ratio of Na^+ is 88% calculated from charge capacity and theoretical capacity.

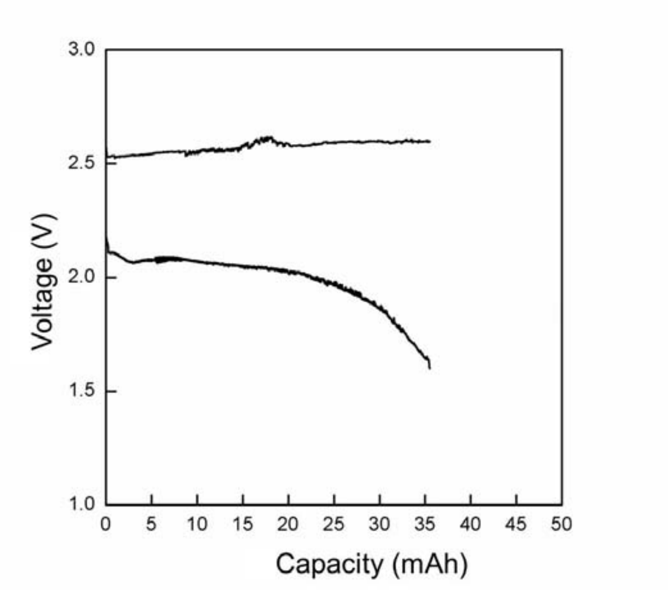

Supplementary Figure 13 | Proof-of-concept of a redox flow battery testing. Typical discharge-charge profile of Na_2S_8|BASE|Na-BP-TEGDME(3 M) redox flow battery with 3 M Na-BP-TEGDME anolyte. Note that the polarization is larger than that of cylinder battery as shown in Fig. 4, which is mainly due to the design of the flow battery: on one hand, the thickness of the BASE plate in the flow battery is 2 mm, however, the thickness of the BASE tube used in the cylinder battery is 1 mm; on the other hand,

the thickness of the electrode in the flow battery is 10 mm while it is only 2 mm in the cylinder battery. We believe that the performance can be further improved by optimizing the system and engineering the cell structure, for instance, using new catholyte system with higher energy density, a thinner Na-β"-Al_2O_3 electrolyte or new electrolyte with higher ionic conductivity, and a highly porous current collector.

Supplementary Tables

Supplementary Table 1 | Total conductivity of Na-BP-DME solutions with different concentrations.

Concentration (mol L^{-1})	0.1	0.2	0.5	1	4
Total conductivity (S cm^{-1})	$2.1x10^{-4}$	$1.7 x10^{-3}$	$6.2 x10^{-3}$	$1.2 x10^{-2}$	$1.7 x10^{-2}$

Supplementary Table 2 | Capacity calculation of Na-BP-DME and Na_2S_8-DMSO

Concentration	Volumetric capacity	Gravimetric capacity

1 M Na-BP-DME (real 0.87 M Na)	23 Ah L^{-1}	25.6 Ah kg^{-1}
2 M Na-BP-DME (real 1.49 M Na)	40 Ah L^{-1}	44 Ah kg^{-1}
3 M Na-BP-DME (real 2.09 M Na)	56 Ah L^{-1}	57 Ah kg^{-1}
4 M Na-BP-DME (real 2.51 M Na)	67 Ah L^{-1}	68 Ah kg^{-1}
5 M Na-BP-DME (real 2.77 M Na)	75 Ah L^{-1}	76 Ah kg^{-1}
Na/Na-BP-DME hybrid anode	1109 Ah L^{-1}	1165 Ah kg^{-1}
1 M Na_2S_8-DMSO	100 Ah L^{-1}	72 Ah kg^{-1}

Volumetric capacity was calculated based on total volume of Na, BP and DME. Gravimetric capacity was calculated based on total mass of Na, BP and DME

Supplementary Table 3 | Energy density calculation

Anode	Cathode	Volumetric	Gravimetric
1 M Na-BP-DME	1 M Na_2S_8-DMSO	42 Wh L^{-1}	42 Wh kg^{-1}
2 M Na-BP-DME	1 M Na_2S_8-DMSO	63 Wh L^{-1}	61 Wh kg^{-1}
3 M Na-BP-DME	1 M Na_2S_8-DMSO	79 Wh L^{-1}	70 Wh kg^{-1}
4 M Na-BP-DME	1 M Na_2S_8-DMSO	88 Wh L^{-1}	77 Wh kg^{-1}
5 M Na-BP-DME	1 M Na_2S_8-DMSO	94 Wh L^{-1}	81 Wh kg^{-1}
Na/Na-BP-DME hybrid anode	1 M Na_2S_8-DMSO	201 Wh L^{-1}	149 Wh kg^{-1}

All of the volumetric energy density calculated based on total volume of anode and cathode, and average operation voltage of 2.2 V. All of the gravimetric energy density calculated based on total mass of anode and cathode, and average operation voltage of 2.2 V.

Supplementary Table 4｜ Cost calculation of Na_2S_8｜BASE｜Na-BP-DME(5 M) 1 kWh cell

Na(\$ 0.23 kg^{-1})	BP(\$ 1.2 kg^{-1})	DME(\$ 1.6 kg^{-1})	Total
0.38 kg	2.53 kg	2.88 kg	5.79 kg
\$ 0.09	\$ 3	\$ 4.6	\$ 7.7

Na_2S(\$ 0.23 kg^{-1})	S(\$ 0.13 kg^{-1})	DMSO(\$ 0.8 kg^{-1})	Total
0.35 kg	1 kg	4.88 kg	6.26 kg
\$ 0.08	\$ 0.13	\$ 3.9	\$ 4.1

Raw material cost of Na_2S_8|BASE|Na-BP-DME(4 M) cell is 11.8 \$ /kWh.

All of these raw materials are non-toxic and environmentally-friendly.

Supplementary Table 5 | Comparison among different battery systems and vanadium RFB.

Battery type	Volumetric energy density	Gravimetric energy density	Reference
Vanadium RFB	25~30 Wh L^{-1}	25~30 Wh kg^{-1}	[1]
AQDS/Br RFB	50 Wh L^{-1}	50 Wh kg^{-1}	[2]
Polymer based RFB	10 Wh L^{-1}	No description	[3]
4-HO-TEMPO/MV RFB	43.2 Wh L^{-1}	No description	[4]
This work	201 Wh L^{-1}	149 Wh kg^{-1}	

Supplementary Methods:

Flow battery construction and electrochemical tests.

A symmetrical flow battery was assembled with Na-BP-TEGDME solutions in both compartments. Nickel foams were used as current collectors, and Na-β"-Al_2O_3 was used as the membrane. In detail, 3 M BP and 3M Na were dissolved in the catholyte, while 3 M BP and 0.5 M Na were dissolved in the anolyte. The volume of each electrolyte was 0.58 mL. The electrolytes were circulated with a peristaltic pump. The charge/discharge test was conducted with a multi-channel potentiostat (Metrohm Autolab, PGSTAT302N) in an argon-filled glove box. The current was 1.0 mA.

A prototype flow battery of Na_2S_8 | BASE | Na-BP-TEGDME was assembled with a piece of Na-β"-Al_2O_3 as the membrane. The catholyte was 0.2 M Na_2S_8 dissolved in

0.5 M $NaSO_3CF_3$-DMSO catholyte, and the anolyte was 3 M Na-BP-TEGDME. The electrolytes were circulated with a peristaltic pump. Charge/discharge test was conducted under a constant current of 0.5 mA with a multi-channel potentiostat (Metrohm Autolab, PGSTAT302N) in an argon-filled glove box.

Chemical titration.

The solubility of Na in the BP-DME solution was determined by a chemical titration. In detail, an excessive Na metal was added into 5 M BP-DME solutions for several days to ensure that the solution was saturated (named as Na saturated BP-DME solution). Then, 1 mL Na saturated BP-DME solution was pipetted to react with 1 mL distill water (note that NaOH was produced in this reaction, which can be titrated by an acid). After complete reaction, the solution was titrated by 0.225 mL HCl (Aldrich, 38% with density of 1.18 g cm^{-3}: 12.3 mol L^{-1}). From this result, we can calculate the solubility of Na in BP-DME is 5 M.

Supplementary References:

1. Li, L., Kim, S., Wang, W., Yang, Z, G., et al. A Stable Vanadium Redox-Flow Battery with High Energy Density for Large-Scale Energy Storage. *Adv. Energy Mater*. **1**, 394-400 (2011).

2. Huskinson, B. *et al.* A metal-free organic-inorganic aqueous flow battery. *Nature* **505**, 195-198

(2014).

3. Janoschka, T. et al. An aqueous, polymer-based redox-flow battery using non-corrosive, safe, and low-cost materials. *Nature* **527**, 78-81 (2015).

4. Liu, T., Wei, X., Nie, Z. et al. A Total Organic Aqueous Redox Flow Battery Employing a Low Cost and Sustainable Methyl Viologen Anolyte and 4-HO-TEMPO Catholyte. *Adv. Energy Mater.* **6**, 1501449(2015).
